# Store-Operated Calcium Entry via STIM1 Contributes to MRGPRX2 Induced Mast Cell Functions

**DOI:** 10.3389/fimmu.2019.03143

**Published:** 2020-01-21

**Authors:** Christopher J. Occhiuto, Ananth K. Kammala, Canchai Yang, Rithvik Nellutla, Marco Garcia, Gregorio Gomez, Hariharan Subramanian

**Affiliations:** ^1^Department of Physiology, Michigan State University, East Lansing, MI, United States; ^2^Department of Pathology, Microbiology and Immunology, School of Medicine, University of South Carolina, Columbia, SC, United States

**Keywords:** mast cells, pseudo-allergic reactions, stromal interaction molecule 1 (STIM1), store-operated calcium entry (SOCE), MAS-related G-protein coupled receptor-X2 (MRGPRX2), MrgprB2

## Abstract

Mast cells are inflammatory immune cells that play an essential role in mediating allergic reactions in humans. It is well-known that mast cell activation is critically regulated by intracellular calcium ion (Ca^2+^) concentrations. MAS-related G-protein coupled receptor-X2 (MRGPRX2) is a G-protein coupled receptor (GPCR) expressed on mast cells that is activated by various ligands, including several FDA approved drugs; consequently, this receptor has been implicated in causing pseudo-allergic reactions in humans. MRGPRX2 activation leads to an increase in intracellular Ca^2+^ levels; however, the Ca^2+^ mobilizing mechanisms utilized by this receptor are largely unknown. Previous reports showed that store-operated Ca^2+^ entry (SOCE) via the calcium sensor, stromal interaction molecule 1 (STIM1), regulates mast cell response induced by the high-affinity IgE receptor (FcεRI). In this study, using complementary pharmacologic and genetic ablation approaches we demonstrate that SOCE through STIM1 promotes MRGPRX2-induced human mast cell response *in vitro*. Importantly, SOCE also critically modulates MrgprB2 (mouse ortholog of human MRGPRX2) dependent inflammation in *in vivo* mouse models of pseudo-allergy. Collectively, our data suggests that MRGPRX2/MrgprB2 activation of mast cells is dependent on SOCE via STIM1, and further characterization of the MRGPRX2-SOCE-STIM1 pathway will lead to the identification of novel targets for the treatment of pseudo-allergic reactions in humans.

## Introduction

Mast cells are innate immune cells with potent inflammatory properties. They express numerous receptors that respond to mechanical and chemical stimuli; their critical function, however, is eliciting allergic responses through crosslinking of the high-affinity IgE receptor (FcεRI). Following FcεRI ligation, mast cells quickly release histamine from pre-stored vesicles during the acute phase of their response through degranulation, leading to the delayed production and release of inflammatory cytokines (1). The IgE receptor is thus imperative in the development of allergic asthma, rhinitis, and food allergies, all of which result from pathogenic hyperactivity of mast cells (2).

MAS-related G-protein coupled receptor-X2 (MRGPRX2) is a recently identified mast cell receptor known to enhance inflammatory responses independent of FcεRI (3–5). This receptor has been implicated in chronic diseases, such as rosacea (6), urticaria (7), atopic dermatitis (8), and rheumatoid arthritis (9). MRGPRX2 displays a promiscuous ligand-binding domain that is potently activated by a variety of different cationic peptides and chemical effectors (10–13). The cathelicidin peptide LL-37 (13, 14), neuropeptide cortistatin-14 (CST-14) (10, 15), and mast cell-degranulating molecule compound 48/80 (3, 16) especially, produce robust degranulation responses through MRGPRX2. LL-37 expression is upregulated in patients with chronic inflammatory skin conditions such as rosacea (17), suggesting that mast cell activation via MRGPRX2 may be responsible for the symptoms associated with this disease. In addition, the pathology of several allergic diseases such as asthma (18) and urticaria (7) correlate with mast cell-specific expression of MRGPRX2. Mouse mast cells express MrgprB2, an ortholog of the human receptor (3). This receptor is activated by the same ligands as MRGPRX2 and displays considerable homology with the human receptor. Interestingly, several FDA approved drugs serve as ligands for MRGPRX2 (3, 19–24) and consequently cause pseudo-allergic reactions in humans; however, the mechanism(s) utilized by this receptor to promote mast cell activation is not well-understood.

Mast cell degranulation is preceded by surges of intracellular Ca^2+^ concentrations. Both FcεRI and MRGPRX2 share similar Ca^2+^ mobilization characteristics (13, 25). Specifically following receptor activation, a rapid increase in intracellular Ca^2+^ is observed that decays very slowly. This sustained Ca^2+^ response induced by MRGPRX2 is surprising, given that MRGPRX2 is a G-protein coupled receptor (GPCR), and most GPCRs induce a transient Ca^2+^ response that almost immediately returns to baseline due to their rapid desensitization and internalization (26). MRGPRX2 displays a prolonged elevation of Ca^2+^ influx comparable to that of the IgE receptor and this response is resistant to desensitization and internalization (13). Mechanistically, FcεRI uses store-operated Ca^2+^ entry (SOCE), which facilitates endoplasmic reticulum (ER)-induced Ca^2+^ flux and Ca^2+^ signal potentiation (27–29). However, it is currently unclear whether MRGPRX2 utilizes SOCE mechanisms for regulating mast cell responses.

The central proponent of SOCE is an endogenous Ca^2+^ sensor called stromal interaction molecule 1 (STIM1) (30). STIM1 contains ER-lumen oriented Ca^2+^-binding domains. Upon cell stimulation and depletion of Ca^2+^ in the ER, these domains become unoccupied, causing a conformational shift and extension of arm-like subunits that complex with calcium release-activated calcium (CRAC) channels and transient receptor potential-canonical (TRPC) channels that are expressed on the cell membrane. Subsequent opening of these channels by STIM1 allows for Ca^2+^ influx into the cytosolic space and increased activation of Ca^2+^-dependent effectors that amplify cellular functions (30, 31).

Since the Ca^2+^-mobilizing machinery utilized by MRGPRX2 in mast cells is unknown, we sought to characterize the Ca^2+^ signaling responses and the proteins involved in MRGPRX2 Ca^2+^ potentiation. Previous studies have demonstrated that FcεRI signaling is largely dependent upon SOCE via STIM1 to facilitate proper mast cell function (29, 32–35). Given the potent inflammatory properties of the IgE receptor and MRGPRX2, in conjunction with their analogous Ca^2+^ mobilization responses, we hypothesized that MRGPRX2 activates SOCE through STIM1. Our data suggests that SOCE via STIM1 is required for MRGPRX2 responses in human mast cells *in vitro*. Additionally, pharmacological inhibition of SOCE attenuated MrgprB2-induced mast cell responses in *in vivo* mouse models of paw edema and rosacea.

## Materials and Methods

### Tissue Culture Media and Reagents

Dulbecco's Modified Eagle's Media (DMEM), penicillin, streptomycin, and L-glutamine supplement were from Corning Cellgro™ (Corning, NY). Recombinant human stem cell factor (hSCF) was purchased from PeproTech (Rocky Hill, NJ). Opti-MEM™ and Stem-Pro™-34 SFM media, puromycin, Lipofectamine® 2000 reagent, and TRIzol™ were purchased from Invitrogen (Carlsbad, CA). Chemical reagents used in buffers, unless otherwise noted, were purchased from Sigma-Aldrich (St. Louis, MO). CST-14 agonist [Pro-c(Cys-Lys-Asn-Phe-Phe-Trp-Lys-Thr-Phe-Ser-Ser-Cys)-Lys], cathelicidin LL-37 (Leu-Leu-Gly-Asp-Phe-Phe-Arg-Lys-Ser-Lys-Glu-Lys-Ile-Gly-Lys-Glu-Phe-Lys-Arg-Ile-Val-Gln-Arg-Ile-Lys-Asp-Phe-Leu-Arg-Asn-Leu-Val-Pro-Arg-Thr-Glu-Ser) and all inhibitors (SKF 96365 HCl (SKF), YM 58483 (YM), A425619, and Nifedipine) were purchased from Tocris Bioscience (Minneapolis, MN). Compound 48/80, substance P and (*R*)-ZINC-3573 were obtained from Sigma-Aldrich. All kits for cDNA synthesis and quantitative PCR were obtained from Applied Biosystems (Foster City, CA). ELISA kits (TNF-α and IL-2) were purchased from BD Biosciences (Franklin Lakes, NJ). Primary western blotting antibodies (anti-STIM1, anti-phospho-p44/42 (p-ERK1/2) anti-p44/42 (T-ERK1/2), anti-phospho-Akt, anti-Akt, and anti-β-actin) were obtained from Cell Signaling Technology (Danvers, MA) and secondary antibodies (donkey anti-rabbit conjugated to IRDye® 680RD and IRDye® 800CW) were purchased from Li-Cor Biosciences (Lincoln, NE). All shRNA plasmids were purchased from Sigma-Aldrich.

### Cells

Human LAD2 cell line was cultured in complete Stem-Pro-34 SFM medium containing penicillin (100 IU/mL), streptomycin (100 μg/mL) and L-glutamine (2 mM) (PSG) supplemented with recombinant hSCF (100 ng/mL) as described by Kirshenbaum et al. (36). Media was hemi-depleted once every week, and cells were maintained at a concentration of 0.8 × 10^6^ cells/mL. HEK-293T human embryonic kidney cells and rat basophilic leukemia (RBL-2H3) cells were obtained from the American Type Culture Collection (Manassas, VA) and were cultured in DMEM supplemented with 10% bovine calf serum and PSG. Cells were split every other day. RBL-2H3 cells stably expressing MRGPRX2 (RBL-MRGPRX2) were generated as described previously (12, 13) and cells expressing the MRGPRX2 receptor were sorted using flow cytometry and phycoerythrin (PE)-conjugated anti-MRGPRX2 antibody (BioLegend, San Diego, CA). The cells were cultured in the DMEM supplemented with 10% bovine calf serum, PSG and G418 (1 mg/mL).

Human skin mast cells were isolated and cultured as described previously (37, 38). Briefly, mast cells were isolated and purified from fresh surgical specimens of human skin tissues that were purchased from the Cooperative Human Tissue Network (CHTN) of the National Cancer Institute. These studies were approved by the human studies Internal Review Board (IRB) of the University of South Carolina. The tissues were mechanically minced and digested with collagenase type II, hyaluronidase, and DNase I in HBSS buffer (1X HBSS, 0.04% NaHCO3, 1% fetal bovine serum, 1% HEPES, 0.1% CaCl_2_). The samples were filtered through 40 μm cell strainers and separated on a Percoll cushion by density centrifugation. The cells at the interface of buffer and Percoll layers were collected, washed, and resuspended at 5 × 10^5^ cells/mL in serum-free X-VIVO 15™ media (Lonza) supplemented with hSCF (100 ng/mL). They were cultured at 37°C 5% CO_2_ with weekly media changes for 8 weeks. Purity was assessed by metachromatic staining with acidic toluidine blue and by flow cytometry staining for FcεRI expression with PE-labeled anti-human FcεRI antibody [clone AER-37 (CRA)] and mouse IgG2bk isotype control (BioLegend). The mast cells were used only when >95% of the cells were FcεRI+ (after ~8 weeks of culture).

### Cell Viability Assays

LAD2 cells (0.5 × 10^5^/well) were plated on 96-well plates and treated with varying concentrations of SKF, YM, Nifedipine, or A425619 for 24 h. The cells were harvested and live and dead cells were counted on a hemocytometer after staining with trypan blue.

### Lentiviral Transduction and STIM1 Knockdown

#### Generation of Lentivirus

Confluent HEK-293T cells (1 × 10^6^ cells) were seeded on to 100 mm^2^ dishes 48 h prior to transfection. Cells were serum-starved for 1 h in Opti-MEM™ medium before addition of Lipofectamine® 2000 reagent and the viral packaging plasmids: p-CMV-VSV-G, pHR'8.2ΔR, and STIM1 shRNA (Cat# SHCLNG-NM_003156; TRC# TRCN0000358718) or scrambled-sequence control shRNA (Cat# SCH002). Media was changed to DMEM after 6 h. Seventy-two hours post-transfection, viral supernatant was harvested, 0.4 μm sterile-filtered, and concentrated using Vivaspin™ protein concentrators (100 kDa MWCO, GE Healthcare).

#### Lentiviral Transduction

LAD2 cells (5 × 10^6^ cells) were washed twice and plated in complete Stem-Pro-34 SFM media supplemented with hSCF (100 ng/mL) and hexadimethrine bromide (polybrene, 4 μg/mL). Concentrated viral supernatant was then added to cells, centrifuged at 700 g for 1 h, and incubated for 8–10 h in 37°C and 5% CO_2_. Media was changed and cells were exposed to puromycin (3 μg/mL) for selection of stable clones and viable cells were used for subsequent experiments.

### Calcium Mobilization

#### SOCE Assay

LAD2 cells were washed and resuspended in 1 mL of 0.1% SIR-BSA (118 mM NaCl, 5 mM KCl, 25 mM HEPES, 5.5 mM glucose, 0.4 mM MgCl_2_, 1 mM CaCl_2_, and 1 mg/mL bovine serum albumin) supplemented with 6 μM Fluo-8 AM calcium dye (Abcam; Cambridge, MA) for 1.5 h at 37°C and 5% CO_2_. Cells were then washed twice with Ca^2+^-depleted 0.1% SIR-BSA and 100 μL (0.3 × 10^6^ cells/mL) were seeded in the same buffer with 0.5 mM EGTA. Using the FlexStation® 3 Flex-protocol, changes in fluorescence were measured for 25 min with the addition of CST-14 and reintroduction of Ca^2+^ at indicated time points. Excitation and emission wavelengths were 490 and 520 nm, respectively.

#### Total Calcium Flux

LAD2 (0.2 × 10^5^ cells in 100 μL) and RBL-2H3 (0.5 × 10^5^ cells in 100 μL) cells were plated in 0.1% SIR-BSA. FLIPR® Calcium 6 dye (Molecular Devices) was reconstituted as per manufacturer's protocol, combined with cells (1:1 ratio) and incubated for 2 h at 37°C and 5% CO_2_. Cells were then stimulated with CST-14, LL-37, substance P, compound 48–80, or (*R*)-Zinc-3573 using the FlexStation® 3 Flex-protocol, and changes in fluorescence were measured over a 120 s period. For assays involving inhibitors, cells were incubated with SKF, YM, Nifedipine, or A425619 for 30 min prior to agonist stimulation. Phosphate-buffered saline (PBS) was used as the control vehicle used for all the inhibitors. Excitation and emission wavelengths were 485 and 525 nm, respectively. Fluorescence data were normalized to maximal response values.

### β-Hexosaminidase Release Assay

LAD2 cells were washed twice and resuspended in 0.1% SIR-BSA. Fourty-five microliters of cells (0.45 × 10^6^ cells/mL) were seeded and stimulated with CST-14, LL-37, substance P, compound 48–80, or (*R*)-Zinc-3573 for 25 min. For total β-hexosaminidase release, cells were lysed using 0.1% Triton X-100. The supernatant (20 μL) was collected and incubated with an equivalent volume of 4 mM p-nitrophenyl-N-acetyl-β-D-glucosamine (PNAG) for 1 h at 37°C. The reactions were halted through the addition of 0.1 M NaHCO_3_/0.1 M Na_2_CO_3_ buffer. The β-hexosaminidase release assay for human skin mast cells was performed as described previously by McHale et al. (39). For assays using inhibitors, cells were incubated for 30 min with the appropriate drug prior to agonist stimulations. Inhibitor concentrations were determined using IC50 values reported by the drug manufacturer. Absorbance was measured using FlexStation® 3 multi-mode plate reader (Molecular Devices; San Jose, CA) at 405 nm. Percent of β-hexosaminidase release content was calculated by dividing absorbances of agonist-stimulated cells by total cell β-hexosaminidase content.

### ELISA

LAD2 cells (0.25 × 10^6^ cells in 250 μL) were washed twice in cytokine-deprived complete Stem-Pro™-34 media, plated and stimulated with corresponding agonists for 6 h. For experiments involving inhibitors, cells were incubated with the appropriate drug for 30 min prior to agonist addition. Cells were then centrifuged and the supernatant was collected. Cytokines (TNF-α and IL-2) in the supernatant was determined by ELISA.

### Western Blotting

Wild type, STIM1 shRNA or scramble (control) shRNA transduced LAD2 cells (4 × 10^6^ cells) were stimulated for different time intervals with LL-37 (3 μM) and lysed using radioimmunoprecipitation assay (RIPA) buffer (150 mM NaCl, 1.0% Triton X-100, 0.5% sodium deoxycholate, 0.1% sodium dodecyl sulfate, 25 mM Tris [pH 8.0], and 5 mM EDTA,) with protease inhibitor cocktail (Roche Applied Sciences; Mannheim, Germany). Fourty micrograms of protein was loaded in a 10% polyacrylamide gel for electrophoretic separation. Proteins were then transferred to nitrocellulose membranes (GE Healthcare). Membranes were blocked in 5% milk solution for 2 h, washed in Tris-buffered saline [pH 7.6] and 0.1% Tween-20 (TBST), then probed with primary antibodies (anti-STIM1, anti-phospho-p44/42, anti-p44/42, anti-phospho-Akt, anti-Akt, and anti-β-actin). The following day, blots were washed in TBST and probed with LiCor IRDye® 680RD or IRDye® 800CW conjugated secondary antibodies for 2 h in the dark. Blots were imaged using LI-COR Odyssey Imaging Systems and analyzed using Image Studio™ Lite (LI-COR Biosciences).

### Mice

C57BL/6 and Balb/c mice were obtained from the Jackson Laboratory (Bar Harbor, ME). All mice were kept under specific pathogen-free conditions. All experiments had the approval of the Institutional Animal Care and Use Committee at Michigan State University. Both male and female mice (6–8 weeks old) were used for experiments.

### Paw Edema Model

C57BL/6 mice were initially treated with PBS (vehicle) or the SOCE inhibitor SKF (30 mg/kg) for 2 days via i.p. injection (3, 22, 40). On the 3rd day, mice received PBS in the right hind paw and compound 48/80 (150 ng in 5 μL) in the left hind paw along with SKF (i.p.). Mice were then injected with 0.15% Evans blue i.v. After 30 min, the thickness of the paws was measured using a micrometer thickness gauge (Peacock thickness gauge, G-1A). The paws were excised, weighed, dried at 50°C, and placed in 1 mL acetone:saline (7:3) for 48 h. The absorbance of the supernatant (250 μL) was quantified at 650 nm.

### Serum Histamine Analysis by LC-MS/MS Method

To estimate the serum histamine levels, we used the method developed by Chimalakonda et al. (41), with a few modifications. After 30 min of compound 48/80 injection in the paws, mice were culled and blood was collected. The serum samples were mixed with three volumes of acetonitrile, vortexed, and centrifuged at 3,000 × g at 4°C for 10 min. The supernatant was dried in a vacuum evaporator and reconstituted in mobile phase (10 mM perfluoroheptanoic acid (PFHA) in water) for LCMS/MS analysis. Histamine standards and spiked serum samples were prepared in mobile phase.

LCMS analysis was performed using a Waters Quattro Micro interfaced with an H-class Waters Acquity UPLC. A Supelco Ascentis Express C18 HPLC column (2.1 × 100 mm, 2.7 μm particle size) was maintained at 40°C in a column oven. The following UPLC-ESI-MS/MS system conditions were used: a mobile phase of solvent A (10 mM PFHA in water) and solvent B (acetonitrile) with gradient elution of solvent B at 10% at time 0 min then ramped to 99% B at 3.50 min, hold at 99% B from 3.50 to 4.50 min and return to 10% acetonitrile at 4.51 min until 6 min with a 0.3 mL min^−1^ flow rate. MS settings were as follows: electrospray ionization in positive ion mode; capillary voltage 2 kV; source temperature 120°C; desolvation temp 350°C; desolvation gas flow 800 L/h. Histamine was monitored using an MRM method with an m/z 112 to 95 transition. Cone voltage was 22 V and collision energy was 16 V with a dwell time of 0.2 s.

### Experimental Rosacea Model

Balb/c mice received i.p. injections of PBS (vehicle) or SKF (30 mg/kg) for 2 days. Mice were then given intradermal injections of LL-37 (50 μL of 320 μM) in their dorsal skin twice a day for 2 days (6) while continuing the SKF treatment. Seventy-two hours after the last LL-37 injection, skin tissues were harvested and either snap-frozen in liquid N_2_ for RNA analysis or fixed in 10% formalin solution for H&E staining. For some experiments, mice were injected with a single dose of LL-37 (50 μL of 320 μM) after the SKF treatments and the skin tissues were harvested and fixed in 10% formalin solution 30 min after the LL-37 injection. Skin inflammation was scored as described previously by Schwartz et al. (42). Briefly, an objective scoring system was employed in a blinded fashion. Erythema, scaling and thickening were scored independently from 0 to 4 as follows: 0, none; 1, slight; 2, moderate; 3, marked; 4, extreme. The average cumulative score of erythema, scaling and thickening served to indicate the inflammation score (scale 0–4). For epidermal thickness measurements, five random epidermal areas in H&E stained skin sections from each mouse were chosen and measured following the acquisition of images using a Nikon® ECLIPSE 50i microscope equipped with a Lumenera® Infinity 3 color camera. For assessing *in vivo* mast cell degranulation, skin tissues were stained with toluidine blue (0.1% in PBS, pH 2.3) and images were captured as described above. Degranulated mast cells (as determined by the staining intensity, appearance, and/or location of the granules) were counted and expressed as percentage of total mast cells in the tissue sections (43).

### Real-Time PCR

Skin samples taken from mice were homogenized in liquid N_2_ using a mortar and pestle. RNA was extracted using TRIzol™ reagent according to the manufacturer's protocol. RNA (2 μg) was transcribed to cDNA using the high capacity cDNA reverse transcription kit from Applied Biosystems. RNA levels (*Ccl2, Il6, Tnf, Mmp9, Tpsab1*, and *Cma1*) were quantified using gene expression assays with TaqMan™ Fast Advanced Master Mix and validated TaqMan™ probes.

### Statistics

Statistical analyses were performed using GraphPad PRISM software (San Diego, CA). A *p*-value less than or equal to 0.05 was deemed to be significant.

## Results

### SOCE Inhibitors Attenuate MRGPRX2 Mediated Ca^2+^ Mobilization, Degranulation, and Cytokine Production in Human Mast Cells

**Figure 1:**
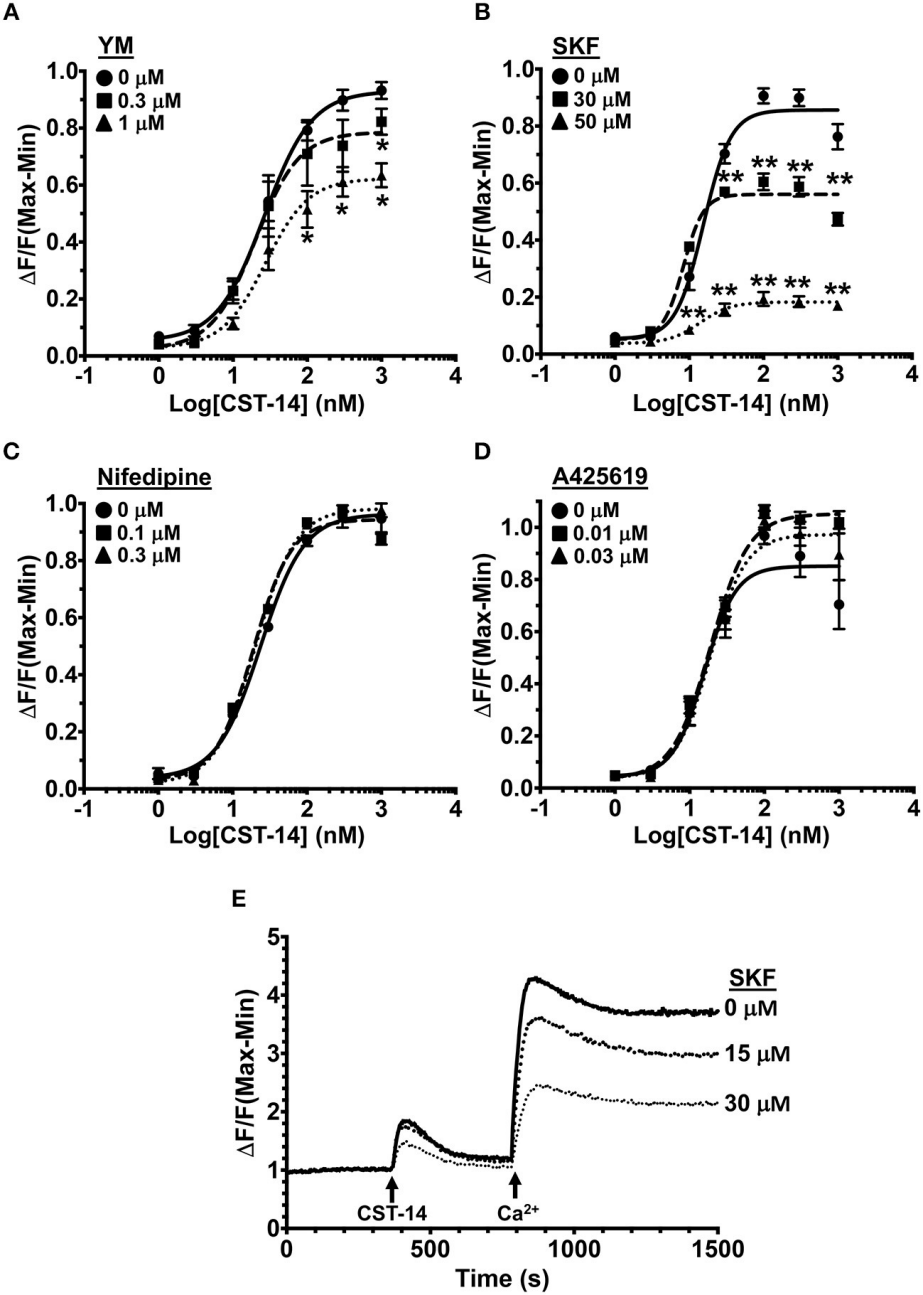
MRGPRX2-induced Ca^2+^ mobilization is reduced by SOCE inhibition. **(A–D)** Intracellular Ca^2+^ mobilization in LAD2 human mast cells was determined following incubation with varying concentrations of **(A)** YM, **(B)** SKF, **(C)** Nifedipine, and **(D)** A425619 for 30 min. Cells were treated with half log doses of the MRGPRX2 agonist cortistatin-14 (CST-14), and changes in fluorescence intensities were recorded for 120 s. Data are plotted as the change in fluorescence [minimum (Min) subtracted from maximum (Max) value] normalized to the maximal change in fluorescence. **(E)** Traces show SOCE assay following SKF pretreatment. LAD2 cells were suspended in Ca^2+^-free buffer and stimulated with 300 nM CST-14. CaCl_2_ (Ca^2+^) at a final concentration of 2 mM was added to the cells at the indicated timepoint. Plotted curves are the average (mean ± S.E.) of 3–6 independent experiments. Data are analyzed with two-way ANOVA. *p < 0.05 and **p < 0.01.

To determine the mechanisms via which MRGPRX2 activation induce Ca^2+^ influx, we activated LAD2 human mast cells with the known MRGPRX2 agonist CST-14 (10) in the presence of drugs that target Ca^2+^ channels and/or Ca^2+^-mobilizing proteins. The doses of the inhibitors were chosen based on the previously reported IC50 values (concentration at which 50% inhibition is observed). The inhibitors did not display any mast cell cytotoxicity at the concentrations used for experiments (Figure S1). Exposure to YM 58483 (YM, IC50 is 0.3–1 μM), an antagonist for calcium release-activated calcium (CRAC) channels (such as ORAI1), significantly decreased the mobilization of intracellular Ca^2+^ (Figure 1A). ORAI channels are activated by the ER Ca^2+^ sensor STIM1 (44, 45). To determine if SOCE via STIM1 contributes to MRGPRX2-induced Ca^2+^ response, we exposed cells to a SOCE inhibitor SKF 96365 HCl (SKF, IC50 is 10–50 μM). A significant dose-dependent reduction in Ca^2+^ influx was observed with SKF suggesting that MRGPRX2-induced Ca^2+^ mobilization is regulated by SOCE via STIM1 and ORAI (Figure 1B). Given the potential off-target effects that SKF may have, we examined the role of voltage-gated Ca^2+^ channels and TRP channels that are known to be susceptible to SKF treatment (46, 47). Nifedipine, an L-type channel antagonist, and A425619, a TRP channel antagonist, did not affect MRGPRX2-induced Ca^2+^ influx (Figures 1C, D) suggesting that SKF indeed targets SOCE via STIM1. Finally, to conclusively verify the role of SOCE, LAD2 cells were placed into Ca^2+^-free buffer and stimulated with CST-14 (Figure 1E). The cells displayed a small, transient increase in fluorescence followed by a rapid return to baseline. Upon re-addition of Ca^2+^ to the buffer, a surge of Ca^2+^-flux into the cell was observed, though SKF diminished this response in a dose-dependent fashion.

**Figure 2:**
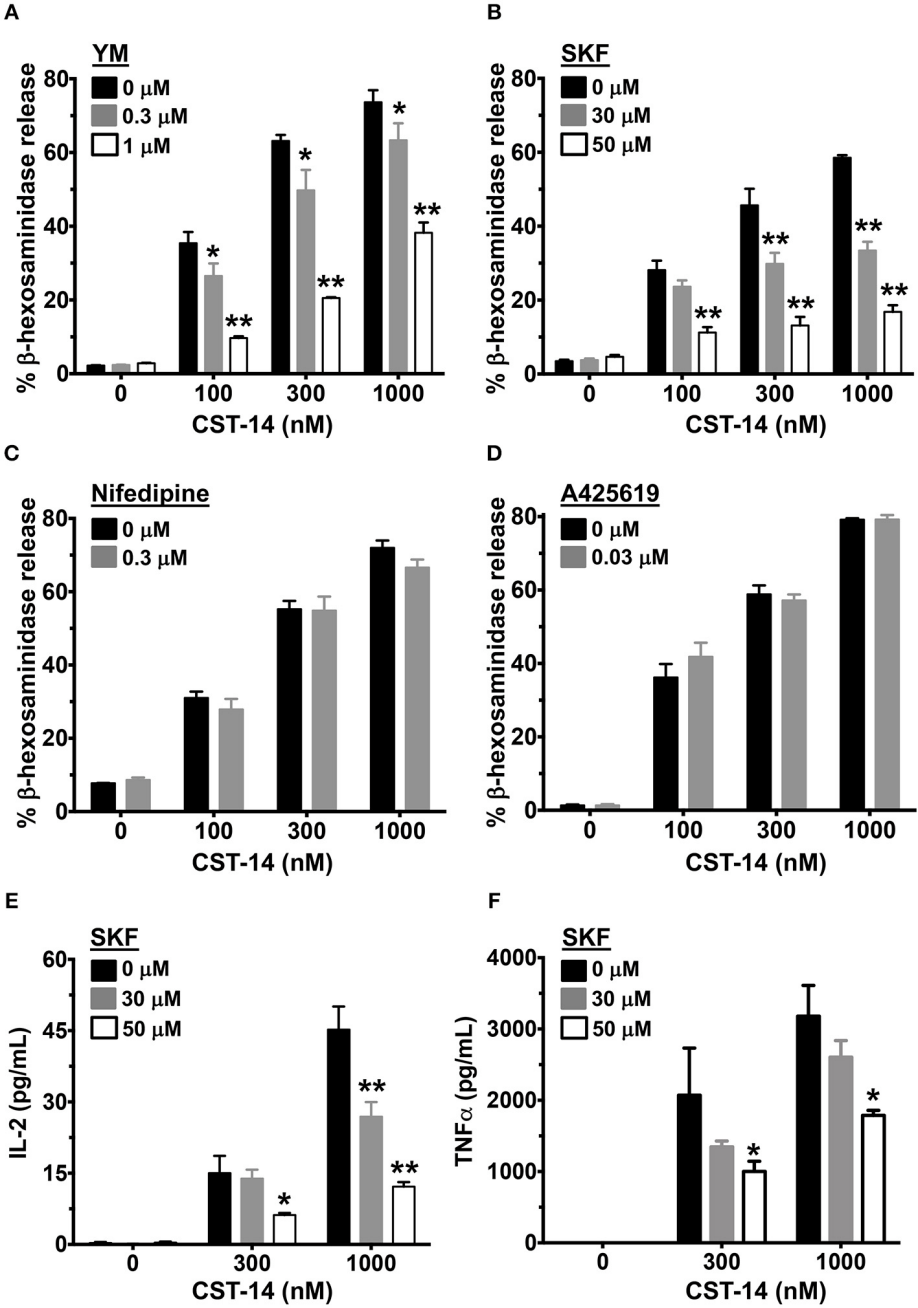
Mast cell degranulation and cytokine production are inhibited by SOCE antagonists. **(A–D)** CST-14-induced degranulation in LAD2 mast cells as quantified by β-hexosaminidase release in the presence of **(A)** YM, **(B)** SKF, **(C)** Nifedipine, and **(D)** A425619 is shown. Values are plotted as percentages of total cell lysate β-hexosaminidase content. **(E,F)** Bar graphs show IL-2 and TNF-α production by LAD2 mast cells stimulated with the indicated concentrations of CST-14. Data shown are mean ± S.E. of 3–5 independent experiments. Statistical significance was determined by two-way ANOVA. *p < 0.05 and **p < 0.01.

Since Ca^2+^ is an important second messenger that regulates the functional responses of mast cells such as degranulation and cytokine production, we analyzed the effects of SOCE inhibition on these mast cell functions. The degranulation response of LAD2 cells (as assessed by the release of β-hexosaminidase) to CST-14 was significantly reduced following pre-treatment with YM and SKF (Figures 2A,B). Consistent with our data in the Ca^2+^ mobilization assays (Figures 1C,D), the L-type Ca^2+^ and TRP channel inhibitors (Nifedipine and A425619) did not have any effect on CST-14-induced mast cell degranulation (Figures 2C,D). These data thus support the role for SOCE via STIM1 and the CRAC channels as the predominant mechanism of Ca^2+^ entry and subsequent mast cell degranulation. Next, we assessed if SOCE regulates delayed mast cell response such as cytokine production following MRGPRX2 stimulation. SKF treatment significantly inhibited the production of IL-2 (Figure 2E) and TNF-α (Figure 2F) in a dose-dependent fashion. Collectively, our data demonstrates that the release of inflammatory mediators by mast cells following MRGPRX2 stimulation is dependent upon Ca^2+^ mobilization through SOCE.

### SKF Inhibits Ca^2+^ Mobilization and Degranulation Induced by Different MRGPRX2 Ligands

**Figure 3:**
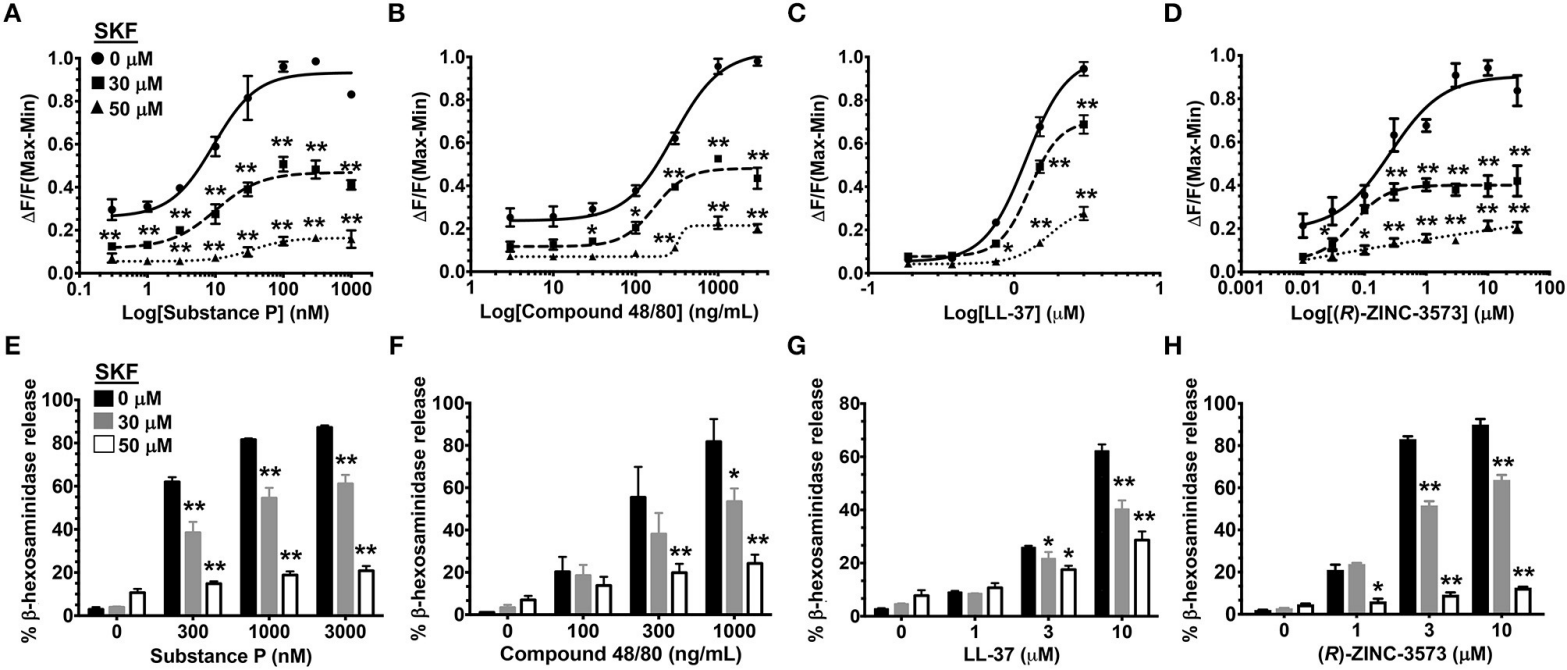
SOCE inhibition attenuates mast cell Ca^2+^ mobilization and degranulation to different MRGPRX2 agonists. LAD2 cells were treated with indicated concentrations of SKF, and Ca^2+^ mobilization **(A–D)** and degranulation **(E–H)** assays were performed following incubation with substance P **(A, E)**, compound 48/80 **(B, F)**, LL-37 **(C, G)** and (*R*)-ZINC-3573 **(D, H)**. Data shown are mean ± S.E. of three independent experiments. Statistical significance was determined by two-way ANOVA. *p < 0.05 and **p < 0.01.

MRGPRX2 is a GPCR that is activated by several ligands that share amphipathic properties (11, 13, 15, 16). As such, the neuropeptide substance P, compound 48/80, and the cathelicidin LL-37 induce potent Ca^2+^ mobilization and mast cell degranulation via MRGPRX2 (3, 13, 16). A recent study (48) identified a synthetic ligand [(*R*)-ZINC-3573] as a potent selective agonist for MRGPRX2. To determine if SOCE is required for MRGPRX2 response induced by these ligands, we exposed LAD2 mast cells to different concentrations of the agonists in the presence of SKF and assessed for Ca^2+^ mobilization and degranulation response. Our data demonstrates that both Ca^2+^ mobilization (Figures 3A–D) and degranulation (Figures 3E–H) in response to substance P, compound 48/80, LL-37 and (*R*)-ZINC-3573 are substantially reduced in the presence of SKF.

RBL-2H3 is a rat basophilic cell line that has been used extensively to assess mast cell activation (49–54). These cells do not endogenously express MRGPRX2 and hence do not respond to CST-14 (16). To determine the specificity of SKF in attenuating MRGPRX2 activation, we generated RBL-2H3 cells stably expressing MRGPRX2 (RBL-MRGPRX2) and sorted cells expressing high levels of this receptor by flow cytometry (Figure S2A). In agreement with previous reports (13, 16, 55), wild type RBL-2H3 (RBL-2H3 WT) cells did not respond to the MRGPRX2 agonists, compound 48/80, substance P, LL-37, and CST-14 for Ca^2+^ mobilization (Figure S2B). However, stable expression of MRGPRX2 rendered these cells responsive to MRGPRX2 stimulation. Consistent with our observation with LAD2 cells, intracellular Ca^2+^ mobilization in RBL-MRGPRX2 cells following stimulation with compound 48/80, substance P, LL-37, and CST-14 was significantly inhibited by SKF (Figure S2B).

### SKF Treatment Affects Mitogen-Activated Protein (MAP) Kinase Activation Following MRGPRX2 Stimulation of Mast Cells

**Figure 4:**
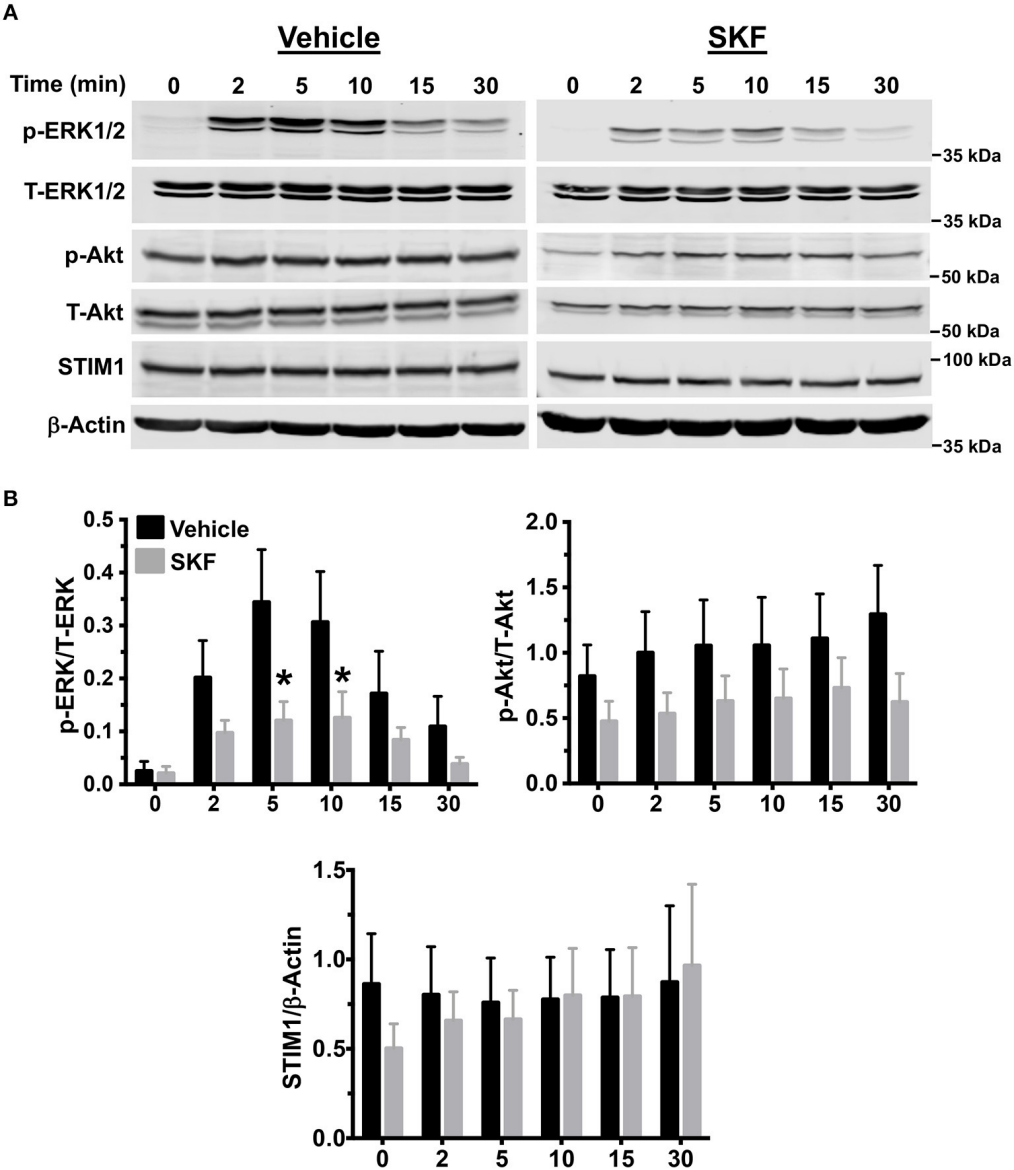
MAP kinase signaling following MRGPRX2 activation is inhibited by SOCE reduction. LAD2 cells were treated with vehicle (PBS) or SKF (30 μM) and exposed to LL-37 (3 μM) for different time intervals. **(A)** Representative western blots are shown. The blots with phosphorylated proteins (p-ERK1/2 and p-Akt) were stripped and reprobed with the total protein (T-ERK1/2 and T-Akt) antibodies. The STIM1 blot was stripped and reprobed with β-Actin (loading control). **(B)** Bar graphs show relative intensities of bands for the indicated signaling proteins. Phosphoproteins were normalized to total expression for each respective target. STIM1 was normalized to β-Actin levels. Data from three independent experiments are shown. Statistical significance was determined by Student's *t*-test. *p < 0.05.

MRGPRX2 activation results in downstream signaling events that activate the MAP kinase and Akt pathways that ultimately regulate mast cell responses such as degranulation and cytokine production (56, 57). Since SOCE regulated mast cell degranulation and cytokine production following MRGPRX2 activation (Figures 2, 3), we examined whether the upstream signaling events were also regulated via the same mechanism. We exposed LAD2 cells to LL-37 for different time intervals, either in the presence or absence of SKF and analyzed for MAP kinase and Akt activation by Western blotting. Transient phosphorylation of the MAP kinase ERK1/2 was evident following LL-37 stimulation, with levels returning to baseline by 30 min. Notably, ERK1/2 phosphorylation was significantly inhibited by treatment with SKF at the 5- and 10-min time points (Figures 4A,B). Contrary to this result, Akt phosphorylation was not altered in the presence of SKF, suggesting that SOCE regulates the MAP kinase but not the Akt pathway following MRGPRX2 stimulation. Additionally, there were no changes in STIM1 expression levels after SKF treatment, indicating that the altered mast cell Ca^2+^ mobilization and functional responses were not due to reduced STIM1 expression following SKF treatment. Taken together, our data suggests that SOCE inhibition following MRGPRX2 activation attenuates Ca^2+^ mobilization and downstream MAP kinase signaling events; thus, diminishing functional responses such as degranulation and cytokine production.

### SKF Inhibits MRGPRX2 Mediated Degranulation of Primary Skin-Derived Human Mast Cells

**Figure 5:**
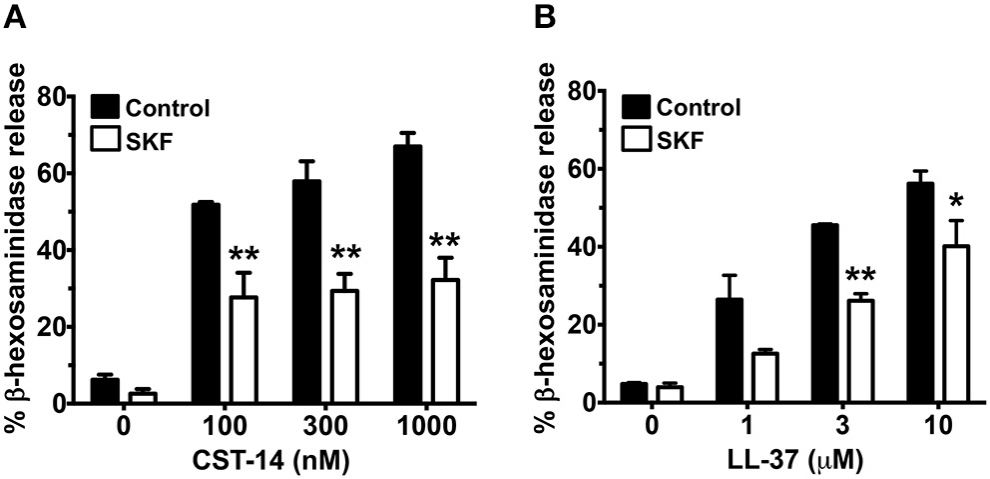
MRGPRX2-induced degranulation of human skin-derived mast cells is attenuated by SOCE inhibition. Human skin-derived mast cells were pre-treated with SKF (50 μM) and exposed to different concentrations of CST-14 **(A)** or LL-37 **(B)**. Bar graphs show degranulation of mast cells as estimated by β-hexosaminidase release in the supernatant. Data shown are mean ± S.E. of three independent experiments with different human donors. Statistical significance was determined by Student's *t*-test. *p < 0.05 and **p < 0.01.

Previous reports have shown that primary human mast cells derived from the peripheral blood as well as human skin mast cells express MRGPRX2 and respond to MRGPRX2 agonists (6, 7, 13). To confirm the biological relevance of our studies obtained with LAD2 cells, we cultured mast cells isolated from the human skin and then exposed these cells to SKF and assessed their degranulation response to different concentrations of CST-14 and LL-37. Consistent with our data from LAD2 cells, SKF treatment significantly reduced degranulation of human skin mast cells to these MRGPRX2 agonists (Figures 5A,B).

### Knockdown of STIM1 Ablates MRGPRX2-Induced Mast Cell Response

**Figure 6:**
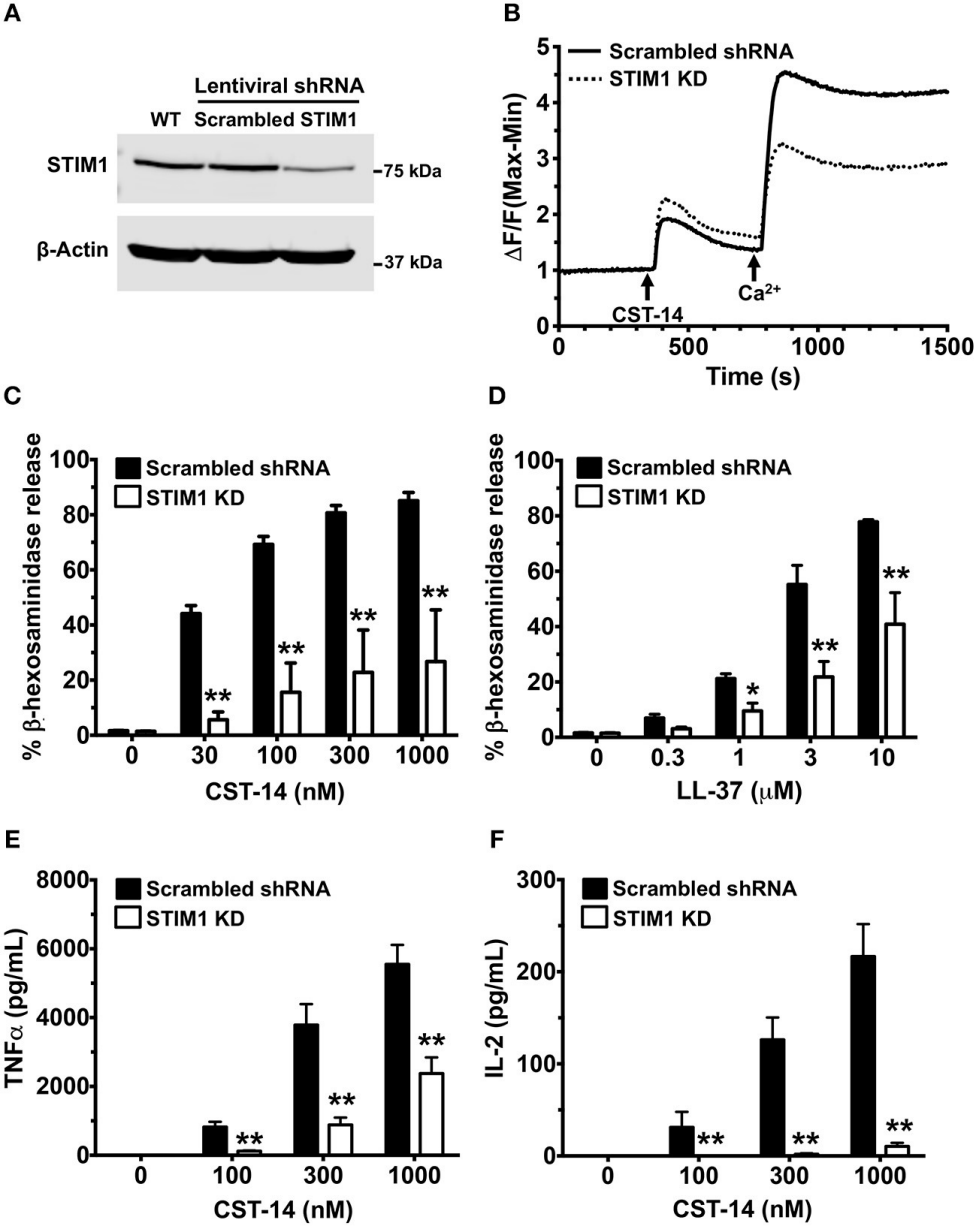
Silencing STIM1 expression inhibits Ca^2+^ mobilization, degranulation and cytokine production in LAD2 mast cells. LAD2 cells were stably transduced with lentivirus containing STIM1 or scrambled shRNA (control). **(A)** A representative blot of STIM1 levels in wild type (WT), scrambled and STIM1 shRNA-transduced cells is shown. **(B)** Control and STIM1 knockdown (KD) cells were labeled with the Calcium 6 dye in Ca^2+^-free buffer. Traces represent the changes in fluorescence following the addition of CST-14 (300 nM) and 2 mM Ca^2+^. **(C,D)** Cells were stimulated with indicated concentrations of CST-14 or LL-37 and β-hexosaminidase release was determined. **(E,F)** Cells were treated with CST-14 for 6 h, and ELISA was performed to estimate TNF-α and IL-2 levels in the supernatants. Data is mean ± S.E. from three experiments. Statistical significance was determined by two-way ANOVA. *p < 0.05 and **p < 0.01.

Pharmacological targeting, in most cases, is prone to off-target effects. To confirm the accuracy of our results obtained with the SKF treatment, we conducted additional experiments where we specifically deleted STIM1 expression in LAD2 mast cells using lentiviral shRNA transduction (12, 13) and performed functional assays with these cells. We used scrambled shRNA transduced cells as controls for our experiments. STIM1 expression levels were reduced (by ~80%) as determined by western blotting (Figure 6A). As expected, SOCE was substantially reduced in STIM1 knockdown cells (Figure 6B). Consistent with the results obtained with SKF and YM (Figures 1–3), degranulation responses to CST-14 (Figure 6C) and LL-37 (Figure 6D) were significantly reduced in cells transduced with STIM1 shRNA, as compared to scramble (control) shRNA-transduced cells. Furthermore, silencing STIM1 expression significantly decreased cytokine production and/or release (Figures 6E,F) with IL-2 (Figure 6F) exhibiting a greater reduction as compared to TNF-α (Figure 6E). These data conclusively demonstrate that SOCE via STIM1 regulates mast cell response following MRGPRX2 activation.

### SKF Administration Attenuates Mast Cell-Dependent Inflammation *in vivo*

**Figure 7:**
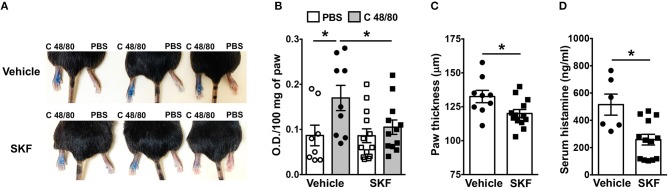
SOCE Inhibition reduces paw edema to compound 48/80. PBS (vehicle) or SKF-treated C57BL/6 mice were exposed to compound 48/80, (C 48/80, left paw) or PBS (right paw) and Evans blue dye was injected i.v. Mice were culled 30 min later. **(A)** Representative pictures of three mice for each group with dye leakage in the paws are shown. **(B)** The paws were excised and weighed; the dye was extracted and absorbance of the supernatant was measured at 650 nm. **(C,D)** Bar graphs shows the paw thickness **(C)** and histamine levels in serum **(D)** of mice. Data shown are mean ± S.E. from three experiments (*n* = 6–13 mice/group). Statistical significance was determined by Student's *t*-test. *p < 0.05.

We next determined if SOCE inhibition affected MRGPRX2-induced responses *in vivo*. Mice express an orthologous receptor to the human MRGPRX2, termed MrgprB2 (3). The mouse receptor is very similar to MRGPRX2; it is activated by the same ligands, facilitates pseudo-allergic phenotypes, and exhibits high sequence homology. We adopted a previously described model of compound 48/80-induced paw edema that is dependent on MrgprB2 expression on mouse mast cells (3). Control vehicle (PBS)- or SKF-treated mice were injected with PBS or compound 48/80 in their right and left paws, respectively, and vascular permeability (an indicator of mast cell degranulation) was assessed following i.v. injection of the Evan blue dye. Paw swelling and histamine levels (an indicator of mast cell degranulation) in the serum were also evaluated in these mice. We observed increased dye extravasation (indicative of vascular leakage), paw swelling and serum histamine levels in the mice treated with compound 48/80, as compared to control PBS treated mice (Figures 7A–D). These responses, however, were significantly reduced in the SKF-treated cohort, suggesting that SOCE inhibition by SKF alleviated compound 48/80-induced paw edema.

**Figure 8:**
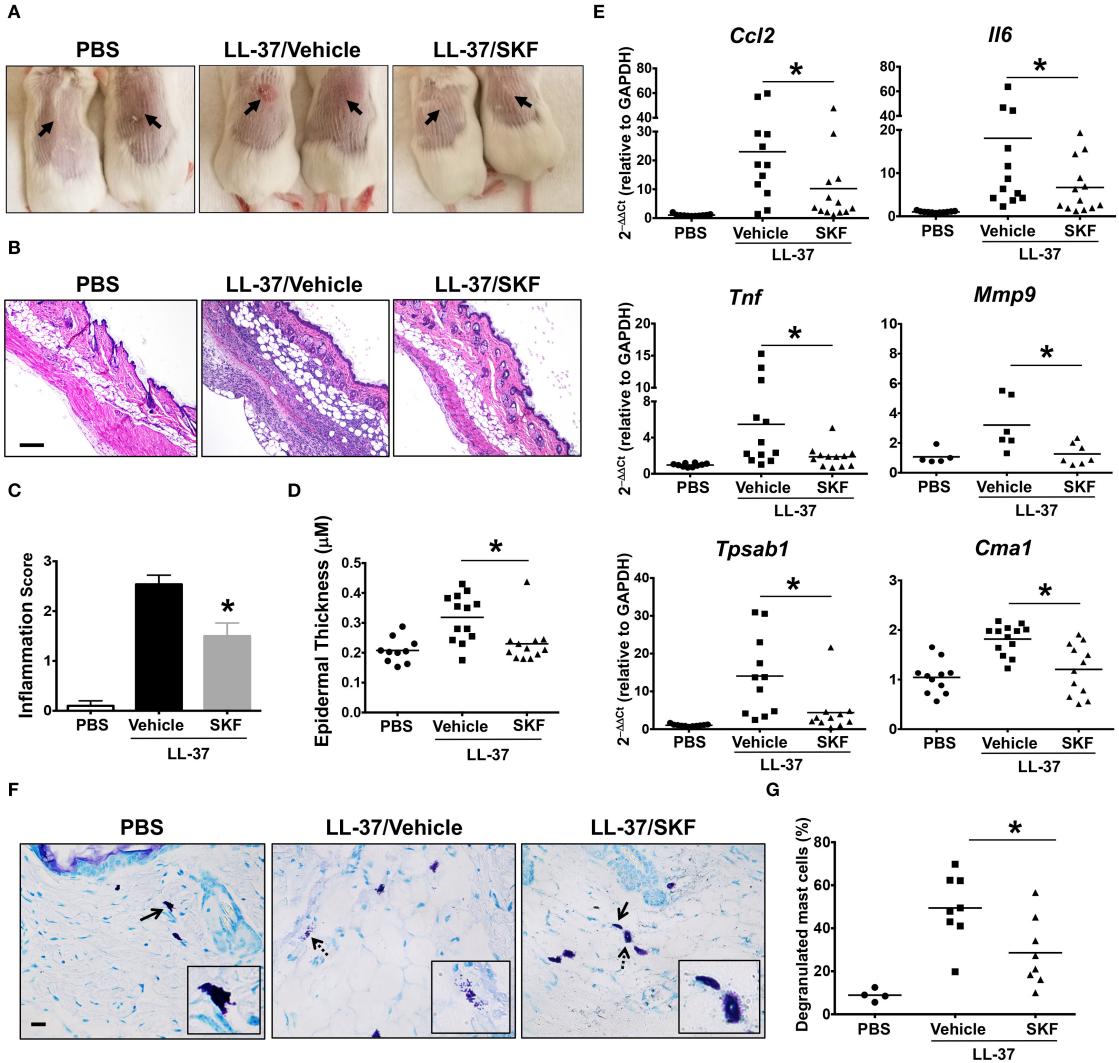
SOCE inhibition prevents the development of LL-37-induced rosacea in mice. PBS (LL-37/Vehicle) or SKF treated (LL-37/SKF) BALB/c mice were injected with LL-37 into the dorsal skin (arrows) twice daily for two consecutive days. Mice that only received PBS on the dorsal skin were used as control. Representative pictures of **(A)** the dorsal skin and **(B)** H&E stained skin sections of mice from different cohorts are shown. Scale bar = 100 μm. **(C,D)** Graphs represent inflammation scores and epidermal thickness of the H&E stained skin sections. **(E)** Relative gene expression of selected gene targets from the excised skin was analyzed by real-time PCR. Values are plotted as 2^−ΔΔCt^ normalized to GAPDH levels. **(F)** Mice were treated with vehicle or SKF and exposed to a single dose of LL-37 in their hind skin. After 30 min of LL-37 injection, the skin tissues were harvested and paraffin embedded skin sections were stained with toludine blue. Representative pictures of the skin sections are shown. Bold arrows indicate intact mast cells whereas dotted arrows represent degranulated mast cells. The inset figure is an enlarged image of the cell(s) shown by the arrows. Scale bar = 50 μm. **(G)** Graph shows the percentage of degranulated mast cells in the skin tissue of different cohorts of mice. Data are mean ± S.E. from three to five experiments (*n* = 4–12 mice/group) Statistical significance was determined by Student's *t*-test. *p < 0.05.

Additionally, we investigated the role of SOCE in a more prolonged and severe skin inflammation model of pseudo-allergic rosacea. Since LL-37 is elevated in the skin tissues of human patients with rosacea, it has been used for inducing the pathogenesis of experimental rosacea in rodents (17, 58). Specifically, Muto et al. (6) showed that the LL-37 injections in the skin caused rosacea like symptoms in mice that are dependent on the presence of mast cells. LL-37-induced Ca^2+^ mobilization and degranulation were attenuated by SKF in human mast cells *in vitro* (Figures 3C,G). To determine whether SKF also inhibits mouse mast cell response *in vivo*, we pretreated mice with PBS (vehicle) or SKF, followed by multiple LL-37 administrations in the dorsal skin. After 72 h, inflammation was evident in the LL-37 treated mice, although, the SKF treated group showed greatly reduced skin reddening and inflammation (Figure 8A). Histological analysis of the skin tissues showed less cellular infiltration and bleeding in the skin of SKF treated mice (Figure 8B) resulting in a significant reduction in inflammation score (Figure 8C) and epidermal thickness (Figure 8D) as compared to the vehicle-treated group. Moreover, RNA analysis of inflammatory markers was consistent with the observed reduction in skin inflammation in the SKF-treated cohort (Figure 8E). Specifically, RNA levels of CCL2, IL-6, TNFα, and MMP9 were significantly decreased in the presence of SKF. Importantly, a significant inhibition in RNA levels of mast cell-associated tryptase (*Tpsab1*) and chymase (*Cma1*) was also observed in the skin of SKF-treated mice. To further examine the role of SKF in attenuating mast cell response *in vivo*, we treated mice with PBS (vehicle) or SKF and exposed them to a single dose of LL-37 in the hind skin. We culled the mice after 30 min of the LL-37 injection and enumerated the numbers of degranulated and non-degranulated mast cells in the skin tissue. Degranulated mast cells exhibited reduced toluidine blue staining intensity and/or dispersed cytoplasmic granules whereas non-degranulated cells were intensely stained and the cytoplasmic granules were not distinctly visible. While there was no difference in the total numbers of mast cells between the PBS and the SKF-treated cohorts of mice (data not shown), the percentage of degranulated mast cells was significantly reduced in the SKF-treated mice as compared to the control vehicle-treated group (Figures 8F,G). Collectively, these data demonstrate that SOCE abrogation by SKF attenuates mast cell-induced inflammation associated with pseudo-allergic reactions *in vivo*.

## Discussion

Ca^2+^ signaling in mast cells remains an important facet of proinflammatory cellular activation. As has been shown for the high-affinity IgE receptor FcεRI (29), STIM1 and SOCE are imperative for the proper Ca^2+^ potentiation and mast cell function. In the current study we examined the role of SOCE as a regulator of MRGPRX2/MrgprB2 mediated responses in mast cells. Our *in vitro* and *in vivo* experiments show that SOCE via STIM1 is an important mechanism for potentiating MRGPRX2/MrgprB2 mediated mast cell activation. This observation is consistent with other receptors implicated in immune activation. Fcε (59), Fcγ (60), as well as T (61), and B cells receptors (62), all appear to utilize SOCE mechanisms when activating their respective cell types.

While analyzing the mechanisms of MRGPRX2-mediated Ca^2+^ mobilization in human mast cells, we observed some residual signaling in both SKF-treated cells and STIM1 knockdown cells. While some of this Ca^2+^ influx may be due to inefficiencies of the SKF drug and the incomplete knockdown of STIM1, respectively, it is important to note that the TRPV4 Ca^2+^ channel has been shown to contribute to MRGPRX2 responses and is subject to upregulation when the cells are stimulated (63, 64). It is therefore possible that some residual Ca^2+^ influx occurs via the TRPV4 channels in the presence of SKF. Activation of TRPV4 channels may be mediated by mechanisms independent of SOCE; however, STIM1 is known to complex with other TRP channels, such as the TRPC subtype and mediate SOCE (65). There may be some degree of activation of TRPV4 by STIM1, although these pathways are yet to be determined. The differential mechanisms utilized by STIM1 and TRPV4 channels to mediate MRGPRX2-induced Ca^2+^ influx and mast cell activation will be the subject of our future investigation.

Mast cell degranulation is promptly initiated by increases of cytosolic Ca^2+^ levels through Ca^2+^-mediated exocytosis. Consistent with our Ca^2+^ mobilization data, SOCE inhibition abrogated most of the degranulation response. Residual degranulation may have been enacted through Ca^2+^-independent signaling pathways or by drawing upon smooth endoplasmic reticulum Ca^2+^ stores. Intracellular signaling events activated by MRGPRX2 are not particularly well-characterized, although, MAP kinase pathways have been shown to be activated in previous studies (56, 66). Our results demonstrated the phosphorylation of ERK1/2 during LL-37 stimulation was significantly reduced in the presence of SKF (Figure 4). An interesting finding of the current study was that activation of Akt was not significantly altered by SKF treatment suggesting that SOCE does not contribute to MRGPRX2-induced Akt activation. MAP kinase and Akt pathways are differentially regulated for different receptors (67) and hence it is possible that while SOCE regulates the MAP kinase pathway for MRGPRX2, a SOCE-independent pathway possibly modulates Akt activation.

In agreement with decreased ERK1/2 activation following SOCE inhibition, LAD2 cells exhibited decreased levels of cytokine production in both SKF-treated and STIM1-silenced cells following MRGPRX2 stimulation indicating that the SOCE-ERK1/2 pathway regulates MRGPRX2 cytokine responses in mast cells. However, it is unclear whether the production of cytokines is being reduced or their release from the cell is affected. Given that Ca^2+^ is important in facilitating a variety of mast cell responses, combined with the decreased ERK1/2 activation in SKF-treated cells, it is conceivable that both the generation as well as the release of cytokines are impaired. LAD2 cells produce copious amounts of pro-inflammatory cytokines such as TNF-α and IL-2 following activation via MRGPRX2. While TNF-α is partially stored in mast cell granules (68), IL-2 is synthesized *de novo* (69). As levels of both of these cytokines are reduced, the notion that both the production and release of cytokines are decreased by abrogating SOCE is strengthened.

Lastly, we demonstrated that paw edema and experimental rosacea were reduced by SKF administration. These models induce pseudo-allergic reactions to MrgprB2 agonists, and this response is mast cell-dependent (3, 6). Because of its short duration and acute nature, the paw edema model corresponds with the immediate phase of mast cell response i.e., degranulation. The accumulation of fluids in the paw tissue is due to histamine released by mast cells following degranulation, which causes subsequent vasodilation and vascular leakage. SKF administration prevented histamine secretion and tissue edema through SOCE inhibition in mast cells, thus complementing our *in vitro* degranulation data. Furthermore, hindering the development of rosacea through SKF injection demonstrates the importance of SOCE in the pseudo-allergic pathology. Although other immune cells may participate in exacerbation of inflammation associated with rosacea, mast cells play a critical role in initiating this disease. We have shown that SFK treatment reduced mast cell Ca^2+^ mobilization and degranulation to LL-37 *in vitro* (Figure 3). Accordingly, the pathology of rosacea was reduced by SOCE inhibition *in vivo*; skin histology showed less inflammatory characteristics, and tissue cytokine/chemokine levels were decreased. Importantly, tryptase, and chymase, two important proteases that are stored and secreted by skin mast cells (70), were reduced in SKF-treated mice. Furthermore, the percentage of degranulated mast cells was also reduced in the skin of mice treated with SKF. These data suggested that mast cell activation was debilitated by SOCE inhibition, and therefore, rosacea pathogenesis was reduced. It also possible that in conjunction with abrogating mast cell response, SKF also affects the inflammatory response of other immune cells involved in rosacea pathology such as T cells. Future studies using mice with mast cell- or T cell-specific STIM1 deletion will determine the contribution of STIM1 expression in these cell types in regulating the rosacea response *in vivo*.

Mast cell MRGPRX2 plays a pivotal role in mediating pseudo-allergic reactions to several FDA approved drugs (3, 19–24) and chronic inflammation associated with asthma (18), urticaria (7), and rosacea (6). We have identified a role for SOCE via STIM1 in regulating MRGPRX2 responses in mast cells. Considering three phases of the mast cell response- immediate Ca^2+^ mobilization, acute degranulation, and latent cytokine production; SOCE inhibition attenuated each of the three steps and reduced inflammation in MRGPRX2 dependent allergic models. Given that SOCE via STIM1 promotes responses of FcεRI (29) and MRGPRX2, two important mast cells receptors that mediate allergy in humans; future studies designed to characterize this mechanism further may lead to the development of novel therapeutic approaches for the treatment of allergic diseases.

## Data Availability Statement

All datasets generated for this study are included in the article/Supplementary Material.

## Ethics Statement

The animal studies were reviewed and approved by Michigan State University's IACUC and Animal Care Program. Studies with human samples were approved by the Internal Review Board (IRB) of the University of South Carolina.

## Author Contributions

CO and AK performed experiments, interpreted data, and wrote parts of the manuscript. CY, RN, MG, and GG performed experiments and analyzed the data. HS conceived the study, planned the experiments, and wrote the manuscript.

### Conflict of Interest

The authors declare that the research was conducted in the absence of any commercial or financial relationships that could be construed as a potential conflict of interest.

## References

[1] Metcalfe DD Baram D Mekori YA Mast cells. Physiol Rev. (1997) 77:1033–79. 10.1152/physrev.1997.77.4.1033 9354811

[2] von Bubnoff D Novak N Kraft S Bieber T. The central role of FcepsilonRI in allergy. Clin Exp Dermatol. (2003) 28:184–7. 10.1046/j.1365-2230.2003.01209.x 12653710

[3] McNeil BD Pundir P Meeker S Han L Undem BJ Kulka M. Identification of a mast-cell-specific receptor crucial for pseudo-allergic drug reactions. Nature. (2015) 519:237–41. 10.1038/nature14022 25517090 PMC4359082

[4] Subramanian H Gupta K Ali H. Roles of Mas-related G protein-coupled receptor X2 on mast cell-mediated host defense, pseudoallergic drug reactions, and chronic inflammatory diseases. J Allergy Clin Immunol. (2016) 138:700–10. 10.1016/j.jaci.2016.04.051 27448446 PMC5014572

[5] Ali H. Emerging roles for MAS-related G protein-coupled receptor-X2 in host defense peptide, opioid, and neuropeptide-mediated inflammatory reactions. Adv Immunol. (2017) 136:123–62. 10.1016/bs.ai.2017.06.002 28950944

[6] Muto Y Wang Z Vanderberghe M Two A Gallo RL Di Nardo A. Mast cells are key mediators of cathelicidin-initiated skin inflammation in rosacea. J Invest Dermatol. (2014) 134:2728–36. 10.1038/jid.2014.222 24844861 PMC4199909

[7] Fujisawa D Kashiwakura J Kita H Kikukawa Y Fujitani Y Sasaki-Sakamoto T. Expression of Mas-related gene X2 on mast cells is upregulated in the skin of patients with severe chronic urticaria. J Allergy Clin Immunol. (2014) 134:622–33.e629. 10.1016/j.jaci.2014.05.004 24954276

[8] Azimi E Reddy VB Lerner EA. Brief communication: MRGPRX2, atopic dermatitis and red man syndrome. Itch. (2017) 2:5. 10.1097/itx.0000000000000005 28367504 PMC5375112

[9] Okamura Y Mishima S Kashiwakura JI Sasaki-Sakamoto T Toyoshima S Kuroda K. The dual regulation of substance P-mediated inflammation via human synovial mast cells in rheumatoid arthritis. Allergol Int. (2017) 66S:S9–20. 10.1016/j.alit.2017.03.002 28366675

[10] Robas N Mead E Fidock M. MrgX2 is a high potency cortistatin receptor expressed in dorsal root ganglion. J Biol Chem. (2003) 278:44400–4. 10.1074/jbc.M302456200 12915402

[11] Subramanian H Kashem SW Collington SJ Qu H Lambris JD Ali H. PMX-53 as a dual CD88 antagonist and an agonist for Mas-related gene 2 (MrgX2) in human mast cells. Mol Pharmacol. (2011) 79:1005–13. 10.1124/mol.111.071472 21441599 PMC3102546

[12] Subramanian H Gupta K Lee D Bayir AK Ahn H Ali H. beta-Defensins activate human mast cells via Mas-related gene X2. J Immunol. (2013) 191:345–52. 10.4049/jimmunol.1300023 23698749 PMC3691353

[13] Subramanian H Gupta K Guo Q Price R Ali H. Mas-related gene X2 (MrgX2) is a novel G protein-coupled receptor for the antimicrobial peptide LL-37 in human mast cells: resistance to receptor phosphorylation, desensitization, and internalization. J Biol Chem. (2011) 286:44739–49. 10.1074/jbc.M111.277152 22069323 PMC3247983

[14] Yu Y Zhang Y Zhang Y Lai Y Chen W Xiao Z. LL-37-induced human mast cell activation through G protein-coupled receptor MrgX2. Int Immunopharmacol. (2017) 49:6–12. 10.1016/j.intimp.2017.05.016 28549244

[15] Tatemoto K Nozaki Y Tsuda R Konno S Tomura K Furuno M. Immunoglobulin E-independent activation of mast cell is mediated by Mrg receptors. Biochem Biophys Res Commun. (2006) 349:1322–8. 10.1016/j.bbrc.2006.08.177 16979137

[16] Kashem SW Subramanian H Collington SJ Magotti P Lambris JD Ali H. G protein coupled receptor specificity for C3a and compound 48/80-induced degranulation in human mast cells: roles of Mas-related genes MrgX1 and MrgX2. Eur J Pharmacol. (2011) 668:299–304. 10.1016/j.ejphar.2011.06.027 21741965 PMC3169012

[17] Reinholz M Ruzicka T Schauber J. Cathelicidin LL-37: an antimicrobial peptide with a role in inflammatory skin disease. Ann Dermatol. (2012) 24:126–35. 10.5021/ad.2012.24.2.126 22577261 PMC3346901

[18] Manorak W Idahosa C Gupta K Roy S Panettieri R Jr Ali H. Upregulation of Mas-related G Protein coupled receptor X2 in asthmatic lung mast cells and its activation by the novel neuropeptide hemokinin-1. Respir Res. (2018) 19:1. 10.1186/s12931-017-0698-3 29295703 PMC5751818

[19] Che D Rui L Cao J Wang J Zhang Y Ding Y. Cisatracurium induces mast cell activation and pseudo-allergic reactions via MRGPRX2. Int Immunopharmacol. (2018) 62:244–50. 10.1016/j.intimp.2018.07.020 30032049

[20] Che D Wang J Ding Y Liu R Cao J Zhang Y. Mivacurium induce mast cell activation and pseudo-allergic reactions via MAS-related G protein coupled receptor-X2. Cell Immunol. (2018) 332:121–8. 10.1016/j.cellimm.2018.08.005 30121125

[21] Navines-Ferrer A Serrano-Candelas E Lafuente A Munoz-Cano R Martin M Gastaminza G. MRGPRX2-mediated mast cell response to drugs used in perioperative procedures and anaesthesia. Sci Rep. (2018) 8:11628. 10.1038/s41598-018-29965-8 30072729 PMC6072780

[22] Zhang T Che D Liu R Han S Wang N Zhan Y. Typical antimicrobials induce mast cell degranulation and anaphylactoid reactions via MRGPRX2 and its murine homologue MRGPRB2. Eur J Immunol. (2017) 47:1949–58. 10.1002/eji.201746951 28688196

[23] Liu R Hu S Zhang Y Che D Cao J Wang J. Mast cell-mediated hypersensitivity to fluoroquinolone is MRGPRX2 dependent. Int Immunopharmacol. (2019) 70:417–27. 10.1016/j.intimp.2019.02.001 30856392

[24] Porebski G Kwiecien K Pawica M Kwitniewski M. Mas-Related G Protein-Coupled Receptor-X2 (MRGPRX2) in Drug Hypersensitivity Reactions. Front Immunol. (2018) 9:3027. 10.3389/fimmu.2018.03027 30619367 PMC6306423

[25] Huber M Helgason CD Damen JE Liu L Humphries RK Krystal G. The src homology 2-containing inositol phosphatase (SHIP) is the gatekeeper of mast cell degranulation. Proc Natl Acad Sci USA. (1998) 95:11330–5. 10.1073/pnas.95.19.11330 9736736 PMC21642

[26] Ferguson SS. Evolving concepts in G protein-coupled receptor endocytosis: the role in receptor desensitization and signaling. Pharmacol Rev. (2001) 53:1–24. Available online at: http://pharmrev.aspetjournals.org/content/53/1/1 11171937

[27] Shaw PJ Feske S. Physiological and pathophysiological functions of SOCE in the immune system. Front Biosci. (2012) 4:2253–68. 10.2741/e540 22202035 PMC3774593

[28] Bergmeier W Weidinger C Zee I Feske S. Emerging roles of store-operated Ca(2)(+) entry through STIM and ORAI proteins in immunity, hemostasis and cancer. Channels. (2013) 7:379–91. 10.4161/chan.24302 23511024 PMC3913761

[29] Baba Y Nishida K Fujii Y Hirano T Hikida M Kurosaki T. Essential function for the calcium sensor STIM1 in mast cell activation and anaphylactic responses. Nat Immunol. (2008) 9:81–8. 10.1038/ni1546 18059272

[30] Smyth JT Hwang SY Tomita T DeHaven WI Mercer JC Putney JW. Activation and regulation of store-operated calcium entry. J Cell Mol Med. (2010) 14:2337–49. 10.1111/j.1582-4934.2010.01168.x 20807283 PMC3074973

[31] Putney JW Steinckwich-Besancon N Numaga-Tomita T Davis FM Desai PN D'Agostin DM. The functions of store-operated calcium channels. Biochim Biophys Acta Mol Cell Res. (2017) 1864:900–6. 10.1016/j.bbamcr.2016.11.028 27913208 PMC5420336

[32] Chen YC Chang YC Chang HA Lin YS Tsao CW Shen MR Differential Ca(2+) mobilization and mast cell degranulation by FcepsilonRI- and GPCR-mediated signaling. Cell Calcium. (2017) 67:31–9. 10.1016/j.ceca.2017.08.002 29029788

[33] Inoh Y Haneda A Tadokoro S Yokawa S Furuno T. Cationic liposomes suppress intracellular calcium ion concentration increase via inhibition of PI3 kinase pathway in mast cells. Biochim Biophys Acta Biomembr. (2017) 1859:2461–6. 10.1016/j.bbamem.2017.09.025 28966111

[34] Yang B Li JJ Cao JJ Yang C-B Liu J Ji Q-M. Polydatin attenuated food allergy via store-operated calcium channels in mast cell. World J Gastroenterol. (2013) 19:3980–9. 10.3748/wjg.v19.i25.3980 23840142 PMC3703184

[35] Ashmole I Duffy SM Leyland ML Bradding P. The contribution of Orai(CRACM)1 and Orai(CRACM)2 channels in store-operated Ca2+ entry and mediator release in human lung mast cells. PLoS ONE. (2013) 8:e74895. 10.1371/journal.pone.0074895 24040356 PMC3769304

[36] Kirshenbaum AS Akin C Wu Y Rottem M Goff JP Beaven MA Characterization of novel stem cell factor responsive human mast cell lines LAD 1 and 2 established from a patient with mast cell sarcoma/leukemia; activation following aggregation of FcepsilonRI or FcgammaRI. Leuk Res. (2003) 27:677–82. 10.1016/S0145-2126(02)00343-0 12801524

[37] Troupin A Shirley D Londono-Renteria B Watson AM McHale C Hall A. A role for human skin mast cells in dengue virus infection and systemic spread. J Immunol. (2016) 197:4382–91. 10.4049/jimmunol.1600846 27799312

[38] McHale C Mohammed Z Gomez G. Human skin-derived mast cells spontaneously secrete several angiogenesis-related factors. Front Immunol. (2019) 10:1445. 10.3389/fimmu.2019.01445 31293594 PMC6603178

[39] McHale C Mohammed Z Deppen J Gomez G. Interleukin-6 potentiates FcepsilonRI-induced PGD2 biosynthesis and induces VEGF from human *in situ*-matured skin mast cells. Biochim Biophys Acta Gen Subj. (2018) 1862:1069–78. 10.1016/j.bbagen.2018.01.020 29410184 PMC5866211

[40] Guo Y Mochizuki T Morii E Kitamura Y Maeyama K. Role of mast cell histamine in the formation of rat paw edema: a microdialysis study. Eur J Pharmacol. (1997) 331:237–43. 10.1016/S0014-2999(97)01002-9 9274985

[41] Chimalakonda KC Pang E Weaver JL Howard KE Patel V Boyne MT 2nd. Development and validation of a liquid-chromatography tandem mass spectrometry method to determine *in vitro* and *in vivo* histamine release. J Pharm Biomed Anal. (2015) 102:494–9. 10.1016/j.jpba.2014.10.016 25459949

[42] Schwartz J Moreno E Calvo A. Combination of paromomycin plus human anti-TNF-alpha antibodies to control the local inflammatory response in BALB/ mice with cutaneous leishmaniasis lesions. J Dermatol Sci. (2018) 92:78–88. 10.1016/j.jdermsci.2018.07.005 30037731

[43] Ngo Nyekel F Pacreau E Benadda S Msallam R Åbrink M Pejler G. Mast cell degranulation exacerbates skin rejection by enhancing neutrophil recruitment. Front Immunol. (2018) 9:2690. 10.3389/fimmu.2018.02690 30515167 PMC6255985

[44] Prakriya M. The molecular physiology of CRAC channels. Immunol Rev. (2009) 231:88–98. 10.1111/j.1600-065X.2009.00820.x 19754891 PMC3253762

[45] Prakriya M Feske S Gwack Y Srikanth S Rao A Hogan PG. Orai1 is an essential pore subunit of the CRAC channel. Nature. (2006) 443:230–3. 10.1038/nature05122 16921383

[46] Chen KH Liu H Yang L Jin MW Li GR. SKF-96365 strongly inhibits voltage-gated sodium current in rat ventricular myocytes. Pflugers Arch. (2015) 467:1227–36. 10.1007/s00424-014-1565-4 25017106

[47] Singh A Hildebrand ME Garcia E Snutch TP. The transient receptor potential channel antagonist SKF96365 is a potent blocker of low-voltage-activated T-type calcium channels. Br J Pharmacol. (2010) 160:1464–75. 10.1111/j.1476-5381.2010.00786.x 20590636 PMC2938817

[48] Lansu K Karpiak J Liu J Huang XP McCorvy JD Kroeze WK. *In silico* design of novel probes for the atypical opioid receptor MRGPRX2. Nat Chem Biol. (2017) 13:529–36. 10.1038/nchembio.2334 28288109 PMC5391270

[49] Maeyama K Hohman RJ Metzger H Beaven MA. Quantitative relationships between aggregation of IgE receptors, generation of intracellular signals, and histamine secretion in rat basophilic leukemia (2H3) cells. Enhanced responses with heavy water. J Biol Chem. (1986) 261:2583–92. 2419319

[50] Kulczycki A Jr. Isersky C Metzger H. The interaction of IgE with rat basophilic leukemia cells. I. Evidence for specific binding of IgE. J Exp Med. (1974) 139:600–16. 10.1084/jem.139.3.600 4812630 PMC2139548

[51] Kulczycki A Jr. Metzger H. The interaction of IgE with rat basophilic leukemia cells. II. Quantitative aspects of the binding reaction. J Exp Med. (1974) 140:1676–95. 10.1084/jem.140.6.1676 4214891 PMC2139755

[52] Robertson D Holowka D Baird B. Cross-linking of immunoglobulin E-receptor complexes induces their interaction with the cytoskeleton of rat basophilic leukemia cells. J Immunol. (1986) 136:4565–72. 2423596

[53] Fewtrell C Metzger H. Larger oligomers of IgE are more effective than dimers in stimulating rat basophilic leukemia cells. J Immunol. (1980) 125:701–10. 7391576

[54] Passante E Frankish N. The RBL-2H3 cell line: its provenance and suitability as a model for the mast cell. Inflamm Res. (2009) 58:737–45. 10.1007/s00011-009-0074-y 19669619

[55] Chompunud Na Ayudhya C Roy S Alkanfari I Ganguly A Ali H. Identification of gain and loss of function missense variants in MRGPRX2's transmembrane and intracellular domains for mast cell activation by substance P. Int J Mol Sci. (2019) 20:E5247. 10.3390/ijms20215247 31652731 PMC6862462

[56] Niyonsaba F Ushio H Hara M Yokoi H Tominaga M Takamori K. Antimicrobial peptides human beta-defensins and cathelicidin LL-37 induce the secretion of a pruritogenic cytokine IL-31 by human mast cells. J Immunol. (2010) 184:3526–34. 10.4049/jimmunol.0900712 20190140

[57] Kiatsurayanon C Niyonsaba F Chieosilapatham P Okumura K Ikeda S Ogawa H. Angiogenic peptide (AG)-30/5C activates human keratinocytes to produce cytokines/chemokines and to migrate and proliferate via MrgX receptors. J Dermatol Sci. (2016) 83:190–9. 10.1016/j.jdermsci.2016.05.006 27237787

[58] Salzer S Kresse S Hirai Y Koglin S Reinholz M Ruzicka T. Cathelicidin peptide LL-37 increases UVB-triggered inflammasome activation: possible implications for rosacea. J Dermatol Sci. (2014) 76:173–9. 10.1016/j.jdermsci.2014.09.002 25306296

[59] Wajdner HE Farrington J Barnard C Peachell PT Schnackenberg CG Marino JP Jr. Orai and TRPC channel characterization in FcepsilonRI-mediated calcium signaling and mediator secretion in human mast cells. Physiol Rep. (2017) 5:e13166. 10.14814/phy2.13166 28292887 PMC5350174

[60] Braun A Gessner JE Varga-Szabo D Syed SN Konrad S Stegner D. STIM1 is essential for Fcgamma receptor activation and autoimmune inflammation. Blood. (2009) 113:1097–104. 10.1182/blood-2008-05-158477 18941110

[61] Lioudyno MI Kozak JA Penna A Safrina O Zhang SL Sen D. Orai1 and STIM1 move to the immunological synapse and are up-regulated during T cell activation. Proc Natl Acad Sci USA. (2008) 105:2011–6. 10.1073/pnas.0706122105 18250319 PMC2538873

[62] Morita T Tanimura A Baba Y Kurosaki T Tojyo Y. A Stim1-dependent, noncapacitative Ca2+-entry pathway is activated by B-cell-receptor stimulation and depletion of Ca2+. J Cell Sci. (2009) 122(Pt 8):1220–8. 10.1242/jcs.041640 19339554

[63] Mascarenhas NL Wang Z Chang YL Di Nardo A. TRPV4 mediates mast cell activation in cathelicidin-induced rosacea inflammation. J Invest Dermatol. (2017) 137:972–5. 10.1016/j.jid.2016.10.046 27908695

[64] Sulk M Seeliger S Aubert J Schwab VD Cevikbas F Rivier M. Distribution and expression of non-neuronal transient receptor potential (TRPV) ion channels in rosacea. J Invest Dermatol. (2012) 132:1253–62. 10.1038/jid.2011.424 22189789 PMC3305847

[65] Worley PF Zeng W Huang GN Yuan JP Kim JY Lee MG. TRPC channels as STIM1-regulated store-operated channels. Cell Calcium. (2007) 42:205–11. 10.1016/j.ceca.2007.03.004 17517433 PMC2764400

[66] Chen X Niyonsaba F Ushio H Nagaoka I Ikeda S Okumura K. Human cathelicidin LL-37 increases vascular permeability in the skin via mast cell activation, and phosphorylates MAP kinases p38 and ERK in mast cells. J Dermatol Sci. (2006) 43:63–6. 10.1016/j.jdermsci.2006.03.001 16600571

[67] Mendoza MC Er EE Blenis J. The Ras-ERK and PI3K-mTOR pathways: cross-talk and compensation. Trends Biochem Sci. (2011) 36:320–8. 10.1016/j.tibs.2011.03.006 21531565 PMC3112285

[68] Olszewski MB Groot AJ Dastych J Knol EF. TNF trafficking to human mast cell granules: mature chain-dependent endocytosis. J Immunol. (2007) 178:5701–9. 10.4049/jimmunol.178.9.5701 17442953

[69] Grutzkau A Kruger-Krasagakes S Baumeister H Schwarz C Kögel H Welker P. Synthesis, storage, and release of vascular endothelial growth factor/vascular permeability factor (VEGF/VPF) by human mast cells: implications for the biological significance of VEGF206. Mol Biol Cell. (1998) 9:875–84. 10.1091/mbc.9.4.875 9529385 PMC25314

[70] Caughey GH. Mast cell proteases as protective and inflammatory mediators. Adv Exp Med Biol. (2011) 716:212–34. 10.1007/978-1-4419-9533-9_12 21713659 PMC3954859

