# Supplementary material for: Store-Operated Calcium Entry via STIM1 Contributes to MRGPRX2 Induced Mast Cell Functions
Source: Front Immunol. 2020 Jan 21;10:3143. doi: 10.3389/fimmu.2019.03143 (PMC6985555; doi:10.3389/fimmu.2019.03143)
Supplement: Supplementary file 1 [file Data_Sheet_1.PDF]

SUPPLEMENTARY FIGURE LEGENDS

Figure S1. Inhibitors of Ca^{2+} channels and/or Ca^{2+} mobilizing proteins have no cytotoxic effects on LAD2 human mast cells. LAD2 cells were incubated with increasing concentration of SKF (0, 30, 100 μM), YM (0, 0.3, 1 μM), Nifedipine (0, 0.1, 0.3 μM), or A425619 (0, 0.01, 0.03 μM) for 24hr. Viable cells were enumerated using trypan blue staining and a hemocytometer. Bar graph shows percent cell viability as compared to PBS treatment (vehicle). The results are expressed as the mean ± S.E. values of 3 independent experiments.

Figure S2. SOCE inhibition attenuates Ca^{2+} mobilization to different MRGPRX2 agonists in RBL-2H3 cells stably expressing MRGPRX2. (A) Histograms show MRGPRX2 expression in wild type RBL-2H3 (RBL-2H3 WT), RBL-2H3 cells stably expressing MRGPRX2 (RBL-2H3 MRGPRX2) and flow sorted RBL-2H3 MRGPRX2 cells. **(B)** Both the RBL-2H3 WT and sorted RBL-2H3 MRGPRX2 cells were treated with vehicle (PBS) or SKF (30 μM), and Ca^{2+} mobilization assays were performed following incubation with compound 48/80 (C 48/80), substance P (Sub P)**,** LL-37 and CST-14. Data shown are mean ± S.E. of 3 independent experiments. Statistical significance was determined by two-way ANOVA. * $p < 0.05$.
