# Supplementary figures and images for: Store-Operated Calcium Entry via STIM1 Contributes to MRGPRX2 Induced Mast Cell Functions
Source: Front Immunol. 2020 Jan 21;10:3143. doi: 10.3389/fimmu.2019.03143 (PMC6985555; doi:10.3389/fimmu.2019.03143)

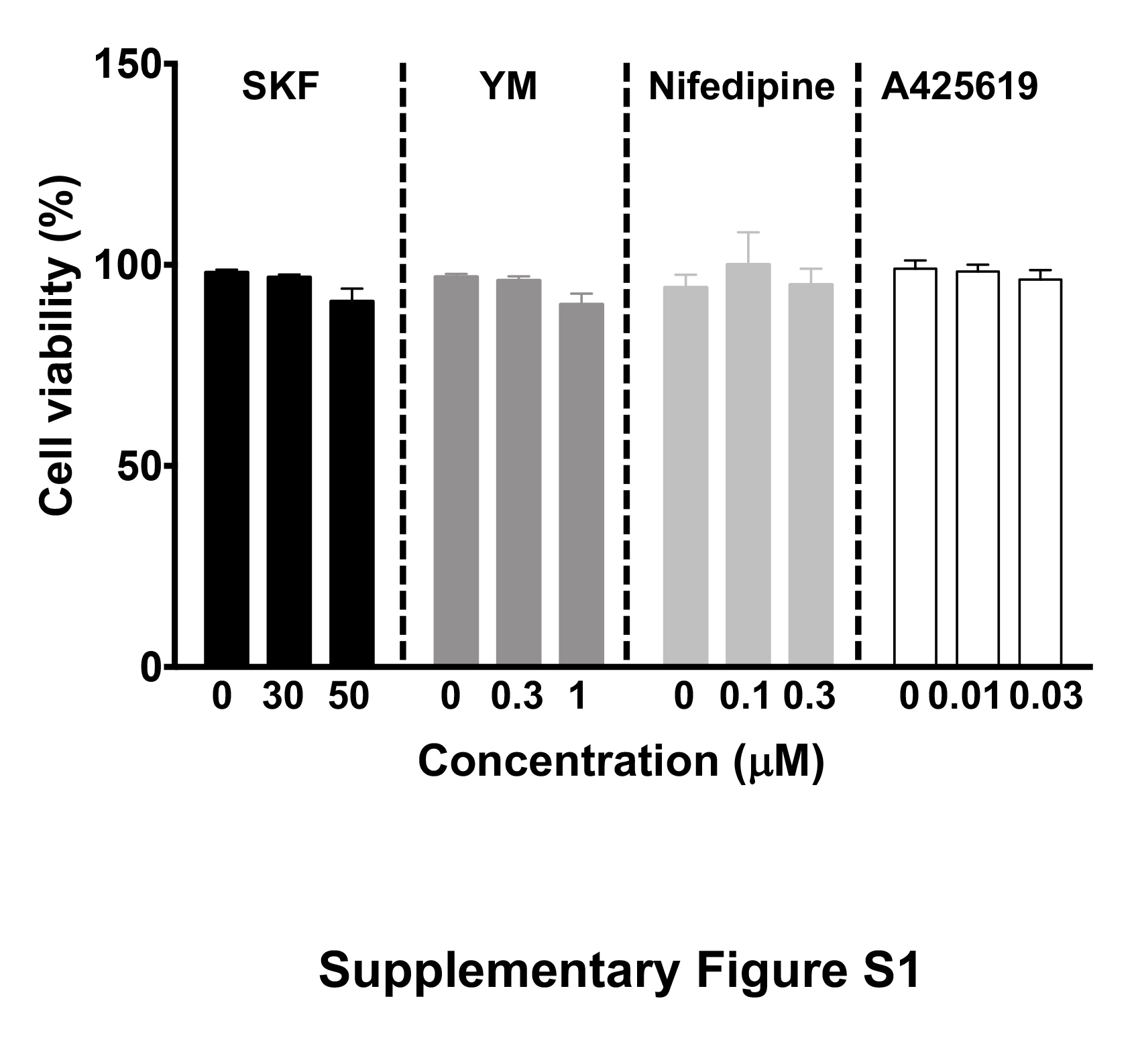

Supplement: Supplementary file 2 [file Image_1.JPEG]

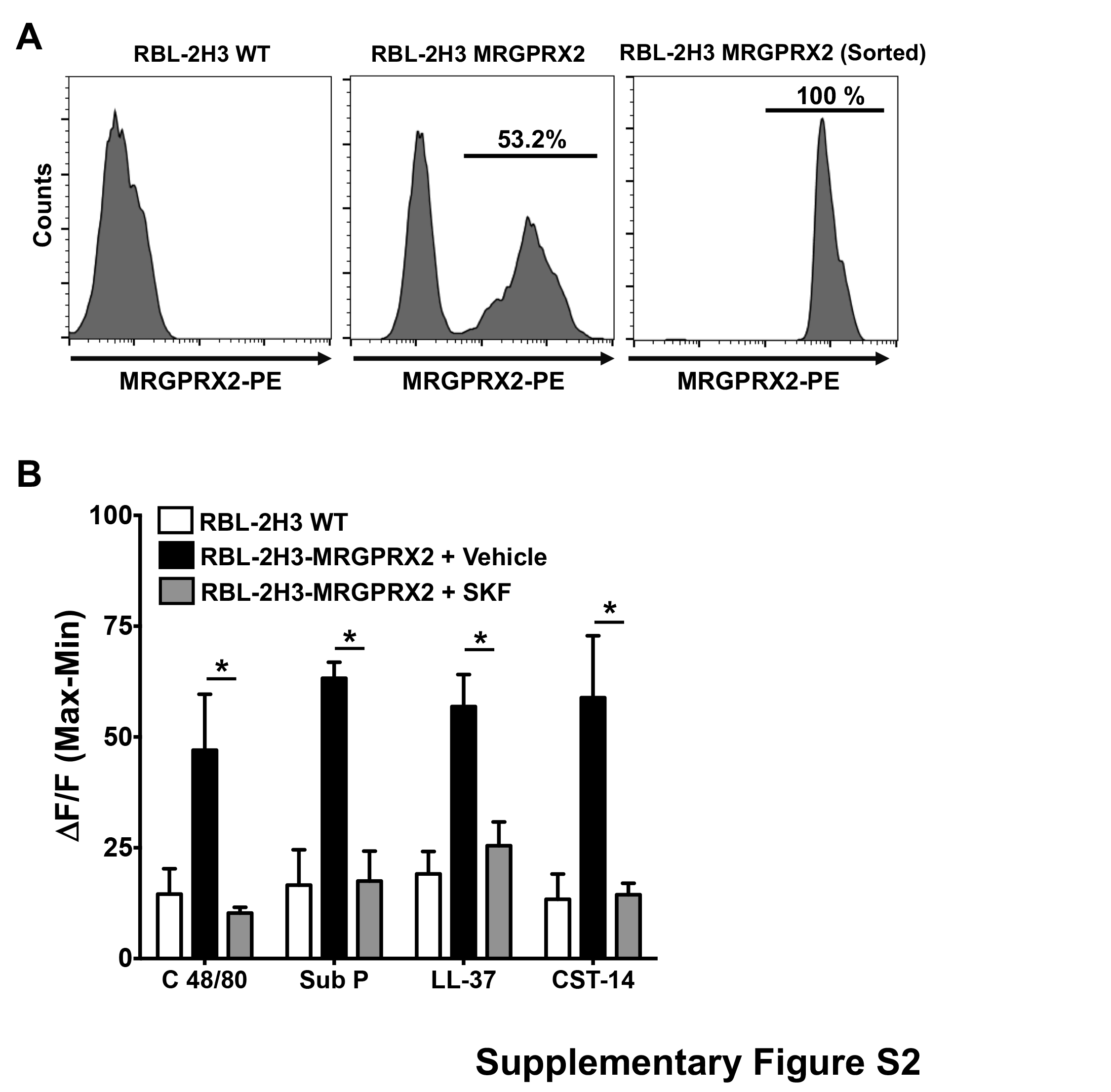

Supplement: Supplementary file 3 [file Image_2.JPEG]
